# Two cases of angular pregnancy with incomplete abortion treated with hysteroscopy: a case report and review of literature

**DOI:** 10.1186/s12893-021-01077-7

**Published:** 2021-02-09

**Authors:** Yin Meichen, Fei Jing, Zhai Lingyun, Zhou Jianwei

**Affiliations:** Department of Gynecology, The Second Affiliated Hospital of Zhejiang University School of Medicine, No88, Jiefang Road, Shangcheng District, Hangzhou, 310002 Zhejiang People’s Republic of China

**Keywords:** Angular pregnancy, Hysteroscopy, Incomplete abortion

## Abstract

**Background:**

Angular pregnancy is characterized as implant medial to the uterotubal junction in lateral angular of uterine. It was a rare obstetric complication with severe complications like uterine rupture and retained placenta.

**Case presentation:**

We report a case of 2 incomplete aborted angular pregnancy that was diagnosed and treated with hysteroscopy. In this case, both of patient were performed operative hysteroscopy for incomplete abortion, and with the assistance of hysteroscopy, the angular pregnancy was detected.

**Conclusions:**

Hysteroscopy can more intuitively display the conditions inside the uterine cavity, reduce the intraoperative and postoperative complications, and shorten the hospitalization time of patients. During hysteroscopy, angular pregnancy can be visualized in the upper lateral side of the uterine cavity. Based on the investigation results of clinical cases, this is the first case report of hysteroscopy in the treatment of incomplete aborted angular pregnancy.

## Background

Angular pregnancy occurs in the following conditions that embryo implants medial to the uterotubal junction and round ligament, while mainly located in the endometrium of the lateral angle of uterus. It was a rare obstetric complication which is first described by the American obstetrician Howard Kelly in 1898 [1]. Unlike interstitial pregnancy, the embryo may develop or miscarry in the uterine cavity. And there are possibilities that angular pregnancy can progress to term. In contrast to normal pregnancy, the placenta in angular pregnancy grows on the restricted, fairly sharp edges of the uterine angle lead to abnormal adhesion of placenta, thickened placenta and muscle weakness. Several life-threatening complications have been reported, including uterine rupture in placenta percreta, retained placenta during vaginal delivery, and hysterectomy due to placenta accreta. In addition to this, taking anatomical factors of uterine angle into consideration, we speculated the increased risk of pregnancy-related complications, including placental abruption, preterm delivery, postpartum endometritis, and growth restriction [2–4]. A meta-analysis supports the view that hysteroscopic resection of products led to a complete removal of products with a lower rate of abnormal complications and intrauterine adhesions compared to blind curettage [5].

A peculiar aspect of these two cases is that we have utilized operative hysteroscopy to remove the incomplete gestational sac located in angular uterine cavity safely and effectively. We present the following cases following the Case REpor (CARE) guidelines.

## Case presentation

### Case 1

**Fig. 1:**
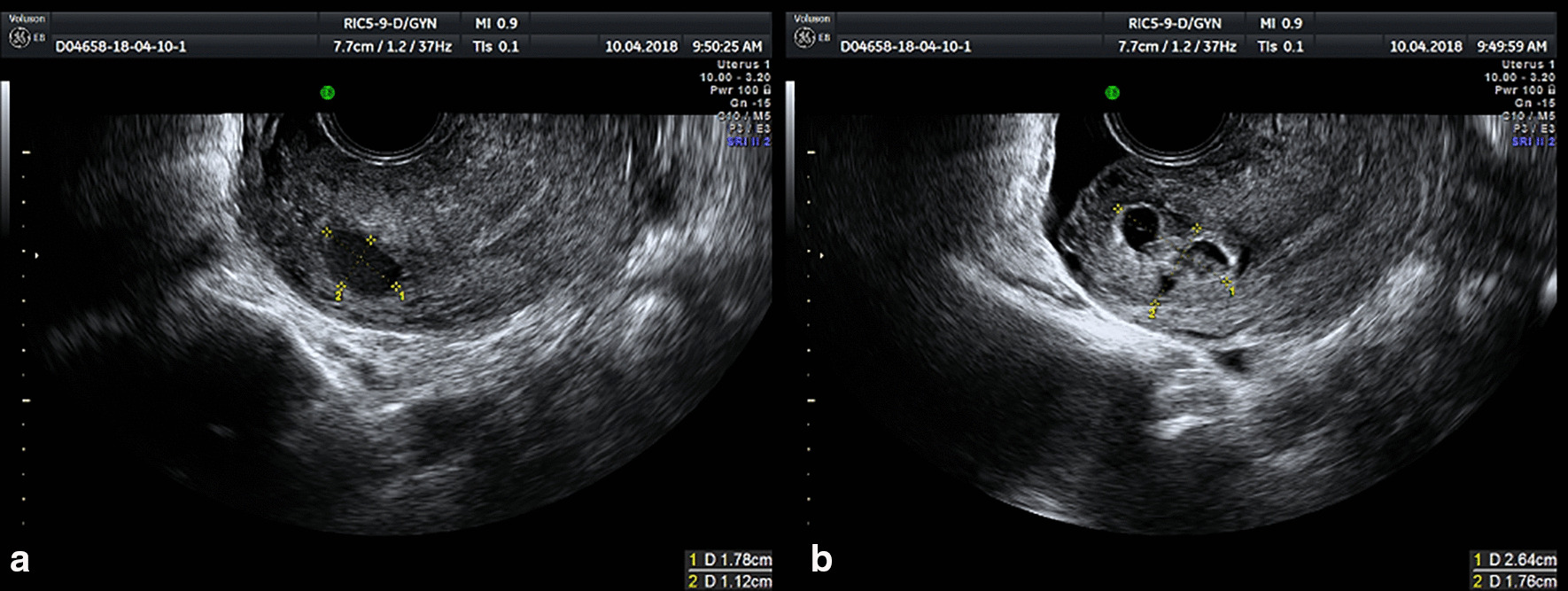
Transvaginal ultrasound image of case 1. A chaotic echo with a range of 2.64 * 1.76 cm in a clear boundary, and a liquid dark area with a range of 1.78 * 1.12 cm were found in right angular of the uterus. Gestation tissue is indicated by measurement of the lesion which looks like yellow dots

**Fig. 2:**
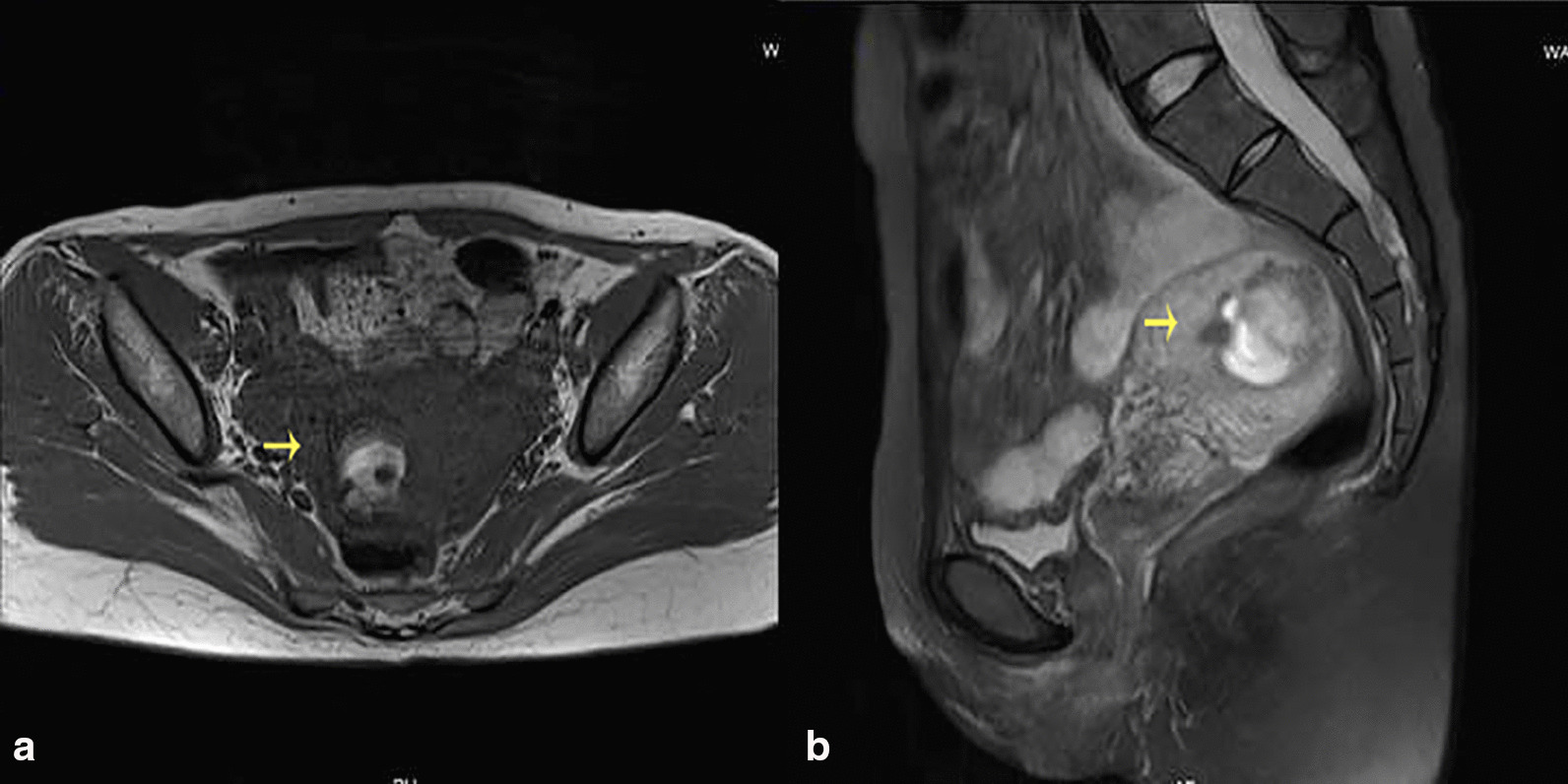
MRI image of case 1. Postoperative image of artificial abortion, mixed signal mass in the right harem of uterus, suggesting the possibility of residual. The arrow points to the gestation tissue

A 34-year-old woman, gravida 3 para 1 at 7 weeks, presented to our hospital for incomplete miscarriage. On April 2, 2018, the patient, diagnosed with early pregnancy, accepted abortion guided with hysteroscopy at Taizhou Boai hospital. However, on the 1 week after surgery, the patient was readmitted to the local hospital due to persistent and massive vaginal bleeding. And Blood HCG tests showed that beta-HCG increased to 32,910 mIU/mL a week post operation and further increased to 40,614 mIU/mL on the 8th day. During these days, patients were regularly reviewed for HCG elevation without special treatment. Several days later, the patient came to our hospital for further treatment, transvaginal ultrasound indicated that mull echo with comparatively prosperous blood flow signals was observed. In the right uterine angle. (Fig. 1) MRI showed there was a visible irregular mixed signal of 30 * 31 * 35 mm occupying the right uterine angular (Fig. 2a, b). Therefore, she was initially diagnosed with induced incomplete abortion accompanying with angular pregnancy. The patient accepted abortion guided by hysteroscope on April 13, 2018. Intraoperatively, we saw abnormal morphology of uterine cavity with dense adhesions in bilateral uterine wall. Right tubal ostium was invisible while filmy adhesions formed in the right cornu, which looked like tubal ostium. When we separated the intrauterine adhesions, the gestational residue was visualized. After suction curettage, some residue was still anchored to the right uterine angle. Finally, the residual pregnancy tissue was excised with hysteroscopic electrode instrumentation, and no residual was found in the uterine cavity. A gross examination fund that intrauterine contents contain chorionic villi. Furthermore, the histological evaluation confirmed the tissue obtained as chorial tissue. Following surgery, beta-HCG was 11,248 mIU/mL on postoperative day 3. After her 20-day of follow-up, beta-HCG reduced to the normal level. There were no procedure-related complications.

### Case 2

**Fig. 3:**
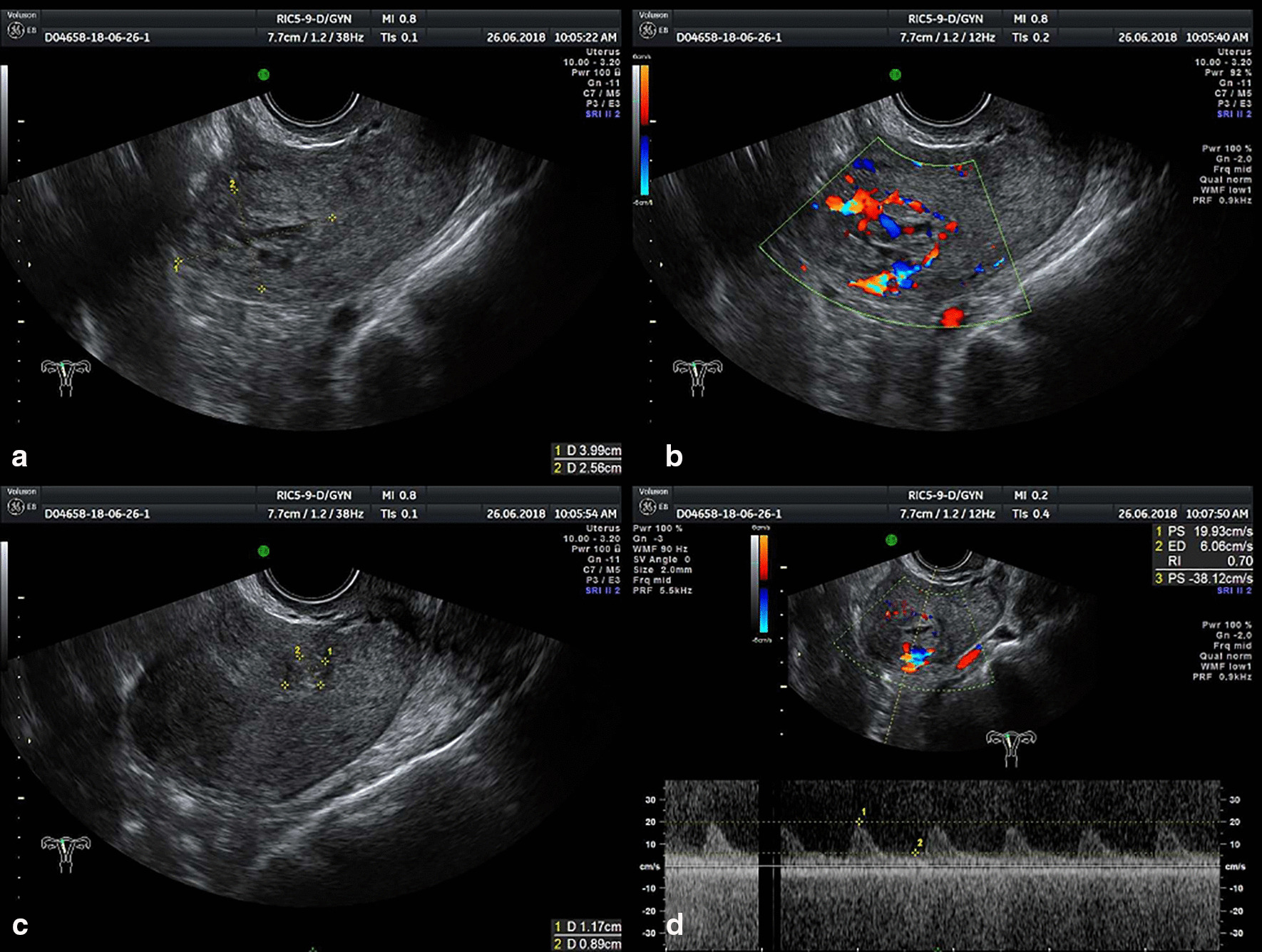
Endovaginal sonography image of case 2. “Honeycomb-resembling” disordered echoes with unclear border located in the left angular of the uterus measuring in 3.99 * 2.56 cm. In figure **a** and **c**, Gestation tissue is marked by yellow dots which was used to measure gestation tissue. In figure **b** and **d**, yellow measurement line circles gestational sac

A woman aged 43 years, gravida 2 para 1 at 5 weeks, has gone through curettage for incomplete miscarriage in Zhejiang Jiangshan hospital. The 4th day after curettage, she appeared to Jiangshan hospital again for postoperative persistent vaginal bleeding. Endovaginal sonography indicated that abnormal echo in the uterus and trophoblastic disease to be excluded. No special treatment was given at the local hospital and the patient later came to our hospital for further treatment. Endovaginal sonography in our hospital demonstrated similar results as local hospital (Fig. 3). Blood β-HCG: 4917.2 mIU/mL. We performed hysteroscope for identifying the intrinsic quality of the abnormal echo in the uterus. During surgery, a 3 * 2 cm villous tissue located in the right angular of the uterus was discovered. And then the villus tissue was carefully excised under hysteroscopy for further examination. Postoperative pathological examination confirmed that villous tissue is decidua. The β-HCG level in the 1st, 2nd and 3rd day after surgery is 13727.2 mIU/mL, 584.4 mIU/mL and 346.9 mIU/mL. Post-operative period was uneventful and this patient discharged from hospital 4 days later.

## Discussion and conclusions

Angular pregnancy is characterized as implant medial to the uterotubal junction in lateral angular of uterine. Angular pregnancy is often confused with cornual pregnancy and interstitial pregnancy. These terms are generally used interchangeably in clinical practice and literature and led to lots of confusions in characterizing the natural course of each entity [1]. Cornual pregnancy refers to a kind of ectopic pregnancy that gestational sac implant in the lateral and upper portion of a rudimentary horn or within one horn of a septate or bicornuate uterus. Interstitial pregnancy is a real ectopic pregnancy which is defined as implantation in the intramural portion of Initial part of tuba-uterine which is covered by myometrial layer. It accounts for approximately 2–11% of tubal ectopic pregnancies and progress asymptomatically beyond the first trimester until uterine rupture followed by hemorrhage at 12–16 weeks pregnancy [6]. The difference between angular pregnancy and interstitial pregnancy is the position relative to the round ligament as seen at operation. The bulging part of interstitial pregnancy is lateral to the round ligament while the enlarged lateral uterus of an angular pregnancy replaces the round ligament reflection outward and upward. Besides, unlike interstitial pregnancy, angular pregnancy are capable of progress to full term. The rate of angular pregnancy ending in abortion was 38.5%, and the incidence of uterine rupture was 23%. Meanwhile, placental accretism is also a common complication of the third trimester in angular pregnancy [7, 8]. Therefore, considering that a portion of the complications is potentially deadly, it seems unwise to treat angular pregnancy as a normal pregnancy with a favorable prognosis. We have searched all cases related to angular pregnancy and their prognosis from pubmed (Tables 1, 2).

**Table 1:** All reported cases of angular pregnancy searched from pubmed

Author	Age	Pregnancy history	GAOAP	Side	Treatment
Chih-Feng Yen et al.	38	G3P1	8 W	Right	Laparoscopy/hysteroscope
Antonio Mollo et al.	34	Unknown	6 W	Left	Multiple-dose methotrexate and hysteroscopy surgery
Denise E. F. Cordeiro et al.	28	G1P1	Unknown	Right	Emergency laparotomy
Katharina Laus et al.	27	G3P1	5 W	Right	Multi-dose methotrexate/diagnostic Laparoscopy/hysteroscope
	32	G1P0	7 W	Right	Laparoscopy/hysteroscope
Malihe Hasanzadeh et al.	34	G4P1	20 2/7 W	Left	Emergency laparotomy
İbrahim Alanbay et al.	34	Unknown	6 W	Right	Cesarean section
Yusuke Tanaka et al.	28	G2P0	6 W	Right	Unknown
Richard B. Mayer et al.	31	Unknown	34 W	Right	Cesarean section/hysterectomy
L. Kambhampati et al.	41	G2P1	6 W	Right	Laparoscopy
Júlio Augusto Gurgel Alves et al.	34	G1P0	6 W	Right	Cesarean section
Ji Young Kwon et al.	37	G3P1	5–7 W	Left	Cesarean section
	22	G1P0	25 4/7 W	Left	Cesarean section
Shashank Shekhar et al.	26	G1P0	30 W	Left	Laparotomy
Baldawa PS et al.	32	G3P1	14 W	Left	Exploratory laparotomy/subtotal hysterectomy/right salpingectomy
Marialuisa Framarino dei Malatesta et al.	32	G1P0	7 W	Left	Transabdominal intralesional injection of methotrexate
	28	G1P0	8 W	Right	Transabdominal intralesional injection of methotrexate
F.-W. Chang et al.	30	Cesarean/bicornuate uterus delivery	9 W	Left	Laparotomy
L. R. BARRON et al.	28	G3P1	14 W	Left	Laparotomy/hysterectomy/oophorectomy
IAN A. MCDOXA et al.	36	G0P0	< 8 W	Left	Laparotomy
LLOYD W. JOHNSTO et al.	31	G2P1	17 W	Right	Subtotal hysterectomy
T. B. FITZGERA et al.	29	G2P1/preterm	41 W	Right	Medical induction/caesarean section
E P RIGBY	Middle-age	G4P3	36 W	Left	Laparotomy/hysterectomy
JAMES T. Louw et al.	27	G3P2	Unknown	Right	Subtotal hysterectomy

*GAOAP* Gestational Age of Confirmed Angular Pregnancy

**Table 2:** Chief complaint, prognosis and complications of all reported clinical cases

Author	PY	Chief complaint	Prognosis	Complications
Chih-Feng Yen et al.	2019	Severe lower abdominal pain	Abortion	No
Antonio Mollo et al.	2018	Pelvic pain	Abortion	No
Denise E. F. Cordeiro et al.	2018	Mild pelvic pain and moderate vaginal bleeding	Abortion	No
Katharina Laus et al.	2018	Abdominal pain	Abortion	No
		Unknown	Abortion	No
Malihe Hasanzadeh et al.	2017	Sudden abdominal pain	Abortion	Rupture of lateral wall of the uterus
İbrahim Alanbay et al.	2016	Unknown	Delivery at 32 W	Preterm
Yusuke Tanaka et al.	2014	Unknown	Delivery at 41 W	No
Richard B. Mayer et al.	2012	Contractions	Delivery at 34 W	Preterm/severe bleeding
L. Kambhampati et al.	2012	Vaginal spotting	Abortion	No
Júlio Augusto Gurgel Alves et al.	2011	Vaginal bleeding	Delivery at 36 W	Preterm
Ji Young Kwon et al.	2011	Vaginal spotting	Delivery at 23 4/7 w	Preterm/placental abruption
		Persistent uterine contraction	Delivery at 25 6/7 w	Preterm
Shashank Shekhar et al.	2010	Preterm	Delivery at 30 W/Hysterotomy	Preterm/retained placenta
Baldawa PS et al.	2008	Hypovolemic shock	Abortion	Rupture of angular pregnancy
Marialuisa Framarino dei Malatesta et al.	2007	Unknown	Abortion	No
		Unknown	Abortion	No
F.-W. Chang et al.	2003	Abdominal spasmodic pain and intermittent vaginal bleeding	Abortion	No
L. R. BARRON et al.	1966	Abdominal pain	Abortion	Ruptures of the uterus
IAN A. MCDOXA et al.	1957	Sudden explosive pain with severe vaginal haemorrhage	Abortion	Perforation of uterus
LLOYD W. JOHNSTO et al.	1952	Lower abdominal pain and vaginal bleeding	Died	Rupture of the uterine wall/gas-gangrene infection of the uterus
T. B. FITZGERA et al.	1952	7 days overdue by her expected date of Confinement	Delivery at full term of gestation	Breech presentation
E P RIGBY	1951	Epigastrium and vomiting associated with pain in the lower abdomen	Fetal death in utero	Ruptures of the uterus
JAMES T. Louw et al.	1947	Tonic contraction of the uterus with constriction ring	Delivery with subtotal hysterectomy	No

*PY* publish year

It is difficult to diagnose angular pregnancy with certainty and hard to judge the exact site of implantation since the gestational sac grows in size. Jansen and Elliott proposed specific criteria for the diagnosis of angular pregnancy in 1981: (1) Clinical presentation with painful asymmetric. (2) enlargement of the uterus. (3) Direct observation of the lateral expansion of the uterus, with or without rupture, accompanied by lateral displacement of the round ligament reflection. (4) Placenta retention at uterine angle. Similarly, diagnosis of angular pregnancy with ultrasound also confronted with many obstacles as round ligament is not displayed via this technique. When ultrasound found a gestational sac.surrounded primarily by the endometrium with a thick, adjacent endometrium, angular pregnancy can be diagnosed. Intravaginal ultrasound can accurately estimate angular pregnancy, especially in early pregnancy. In 2-D sonogram, an angular pregnancy was highly suspected when the sac is easily visualized with the probe tilted and rotated towards the uterine angle. 3-D sonogram expert in offering scene of uterus that can not be acquired with 2-D sonography. In 3-D sonogram visualizations of angular pregnancy, the sagittal plane of the uterus clearly shows that the gestational sac is shifted to the upper lateral border of the uterine cavity. The uterine angle at this site is enlarged, and the myometrium is thinner than the contralateral uterine myometrium. Three-dimensional ultrasound and magnetic resonance are of great significance in the diagnosis of angular pregnancy, reduce the possibility of misdiagnosis, assess abnormal placental implantation and predict the risk of uterine rupture [9]. As shown in our cases, these two patients had not been clearly diagnosed with angular pregnancy prior to hysteroscopy, and their surgical indications were incomplete miscarriages.

Hysteroscopy is considered the gold standard procedure for diagnosing and treating uterine cavity pathologies because it provides the opportunity to directly visualize/biopsy and simultaneously treat pathology. Hysteroscopic surgery is an alternative method of uterine curettage, which shows the advantages of "visual control", thereby improving complete treatment by limiting the field of surgery, reducing the second procedure rate and intrauterine adhesion. Hysteroscope, directly observe the uterine cavity through the cervix under the endoscope and CO_2_ gas insufflation. During hysteroscopy, angular pregnancy can be visualized in the upper lateral side of the uterine cavity [10]. Our cases fully demonstrate the significance of hysteroscopy in the diagnosis and treatment of angular pregnancy. Because both of the patients underwent an uneventful postoperative period and discharged from the hospital without any complications.

In conclusion, this is the first case talking about hysteroscopy in diagnosis and treatment of incomplete aborted angular pregnancy, especially in the diagnosis of angular pregnancy. Meanwhile, this case gives us such a revelation: when the patient presents with clinical symptoms of incomplete abortion, we must pay attention to the implantation site of the pregnancy sac, entirely exclude angular pregnancy and other ectopic pregnancy, to avoid misdiagnosis.

## Data Availability

The datasets supporting the conclusions of this article are included within the article.

## Abbreviation

HCG: Human chorionic gonadotropin
